# Pharmacologic Properties of the Carrier Solutions for Hyperthermic Intraperitoneal Chemotherapy: Comparative Analyses Between Water and Lipid Carrier Solutions in the Rat Model

**DOI:** 10.1245/s10434-018-6628-x

**Published:** 2018-07-18

**Authors:** Eun Jung Park, Junhyun Ahn, Sang Won Gwak, Kyung Su Park, Seung Hyuk Baik, Sung-Joo Hwang

**Affiliations:** 1 Division of Colon and Rectal Surgery, Department of Surgery, Gangnam Severance Hospital, Yonsei University College of Medicine, Seoul, Korea; 2 Yonsei Institute of Pharmaceutical Sciences, Yonsei University, Incheon, Korea; 3 College of Pharmacy, Yonsei University, Incheon, Korea; 4 Advanced Analysis Center, Korea Institute of Science and Technology, Seoul, Korea

## Abstract

**Background:**

Carrier solutions play an important role in the distribution, plasma absorption, chemical stability, and solubility of anticancer agents during hyperthermic intraperitoneal chemotherapy (HIPEC). In the current study, lipophilic properties of carrier solutions were evaluated to determine whether they improved anticancer drug absorption rates using mitomycin-C (MMC) or oxaliplatin HIPEC as compared to hydrophilic carrier solutions.

**Methods:**

Sprague-Dawley rats were divided into two groups: MMC and oxaliplatin treatment groups. Each group was then further subdivided by carrier solution: Dianeal^®^ PD-2 peritoneal dialysis solution, 5% dextrose solution and 20% lipid solution (Lipision^®^). HIPEC was performed over 60 min at 41–42 °C using the anticancer drugs MMC (35 mg/m^2^) or oxaliplatin (460 mg/m^2^). The plasma area under the curve (AUC; AUC_plasma_), peritoneal AUC (AUC_peritoneum_), and peritoneal/plasma AUC ratios were compared among HIPEC carrier solutions.

**Results:**

Plasma drug concentrations were significantly different among carrier solutions, varying by time. In contrast, peritoneal drug concentrations did not change with carrier solution. In the MMC group, the peritoneal/plasma AUC ratio of a lipid solution was three times higher than Dianeal^®^ (*p* < 0.001). In the oxaliplatin group, the peritoneal/plasma AUC ratio was significantly different between carrier solutions (*p* = 0.046). Although the oxaliplatin AUC_peritoneum_ did not vary (*p* = 0.941), the AUC_plasma_ of a lipid solution was lower than that of 5% dextrose solution (*p* = 0.039).

**Conclusions:**

The lipid carrier solution increases the peritoneal/plasma AUC ratio and decreases plasma absorption rates. However, further study is required before clinical uses, considering its pharmacologic properties and possible risks after HIPEC.

**Electronic supplementary material:**

The online version of this article (10.1245/s10434-018-6628-x) contains supplementary material, which is available to authorized users.

Hyperthermic intraperitoneal chemotherapy (HIPEC) after cytoreductive surgery is utilized based on studies showing that microscopic residual tumors can be eradicated by intraperitoneally (IP)-administrated anticancer drugs, with enhanced cytotoxic effect at 41–43 °C.^1^,^2^ As IP drug administration results in a high concentration gradient in the peritoneal-plasma barriers, anticancer drugs can infiltrate tumors following the principles of convection, diffusion, and recirculation, which is different from systemic chemotherapy administration.^1^,^3^,^4^ High drug concentration within the peritoneal cavity produces a driving force for anticancer drugs to infiltrate tumor cells during HIPEC.^3^ In addition, slow plasma absorption rates of anticancer drugs enhance IP drug efficacy and reduce systemic toxicities.^5^

Anticancer drug tissue penetration during HIPEC depends on several pharmacologic properties, including drug concentration, time of exposure, molecular weight (MW), temperature, and lipophilicity.^6^ For optimal HIPEC treatment, it is crucial to select the appropriate carrier solution for each anticancer drug to enhance drug activity, because pharmacologic properties are uniquely different during use in the peritoneal cavity.^6^,^7^ Especially, carrier solutions have an important role in the distribution, plasma absorption, chemical stability, and solubility of anticancer agents during HIPEC.^8^,^9^ Previous reports evaluated hydrophilic carrier solution pharmacologic properties for HIPEC.^6^,^10^–^12^ In the current study, the ability of lipophilic carrier solutions to improve the absorption rate of anticancer drugs during HIPEC was determined. In addition, the pharmacologic characteristics of mitomycin C (MMC) and oxaliplatin were evaluated according to HIPEC carrier solution to find the optimal pharmacologic conditions to treat patients with colorectal cancer carcinomatosis.

## Methods

### Experimental Design

According to anticancer treatment, animals were divided into two groups: MMC or oxaliplatin HIPEC. Three carrier solutions were evaluated in this study: Dianeal^®^ PD-2 1.5% peritoneal dialysis solution (Baxter, USA), 5% dextrose solution, and 20% lipid solution (Lipision^®^, JW Pharmaceutical, Republic of Korea). Three rats were used to perform HIPEC for each carrier solution. The Animal Research Committee of Ajou University, Republic of Korea (IACUC Number 2016-0029) approved the study protocol. Experiments were performed at the Laboratory Animal Research Center at Ajou University Medical Center, Suwon, Korea.

### Animals

Fifteen, 8-week-old, male Sprague-Dawley rats, weighing 290–320 g, were purchased from Orientbio Inc. (Kyunggi-do, Korea). Mean body surface area (BSA) was calculated by using the Du Bois method.^13^ Rats were housed in filter-top cages for 1 week before experiments with free access to food and water (Ziegler^®^ lab animal diet, USA). The animal laboratory was kept under standard conditions with temperature 21–24 °C, humidity 40–60%, 12-h light cycle, and filtered air. A total of 15 rats, comprising 3 rats per group, was used in this study.

### Experimental Settings for HIPEC

**Fig. 1:**
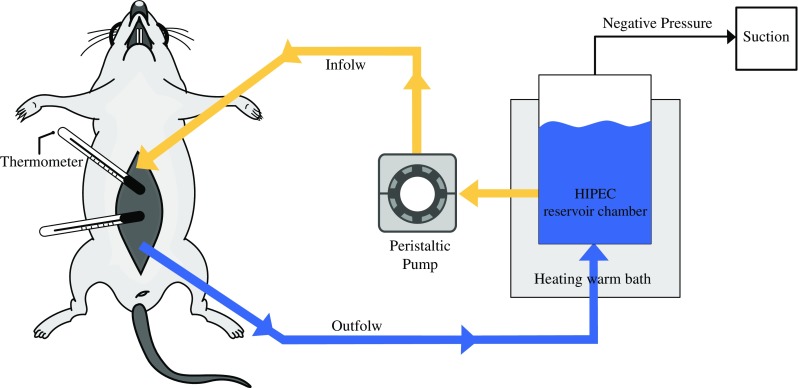
HIPEC rat model

The HIPEC experimental equipment was setup as shown in Fig. 1. The inflow line was inserted into the roller of a peristaltic pump (Masterflex C/L pump^®^, Bernant, USA) and delivered HIPEC solutions at a flow rate of 40 mL/min. The outflow line connected the abdominal cavity to a reservoir chamber. Outflow line circulating fluids could be returned to the reservoir chamber using negative pressure induced by suction. The temperature of circulating HIPEC solutions was maintained at 41–42 °C. Both inflow and outflow lines were heated by a circulating warm bath (Lauda E100^®^, Lauda, Germany). Three sites were monitored for consistent temperature using thermometers: circulating HIPEC solutions in the rat abdominal cavity and rectum, and the heated water in the warm bath.

### Hyperthermic Intraperitoneal Chemotherapy (HIPEC) Procedure

All animals received general inhalation anesthesia using 3% isoflurane with 1:1 oxygen and nitrous oxide before HIPEC procedures. Before the anesthesia, 20 mL of water was given orally to all rats to prevent dehydration. According to the Coliseum technique, a 4- to 5-cm medial longitudinal incision was made in the rat abdominal wall.^14^ Then, all margins of the abdominal wall were elevated and fixed in the acryl plates, which were located 15-cm above the basal plate. After setting the HIPEC equipment as described in Fig. 1, HIPEC solutions were prepared with MMC (35 mg/m^2^) or oxaliplatin of (460 mg/m^2^), which were mixed with 300 mL of carrier solution. Blood and peritoneal fluid samples were collected at 0 (starting time), 5, 10, 20, 30, 45, and 60 min after beginning HIPEC. Peritoneal fluid was collected in the HIPEC circulated fluid of the abdominal cavity, and blood samples were collected from the retro-orbital venous sinus after inhalation anesthesia. All samples were kept frozen at − 60 °C until further analyses.

### Sample Preparation and Analytical Methods

Samples were thawed at room temperature before analyzing drug concentration. Protein precipitation was performed to remove blood and peritoneal components. For MMC analysis, samples were centrifuged at 15,000 rpm at 4 °C for 15 min. Then, 75 μL of the supernatant was diluted with 90% acetonitrile (300 μL). After vortex shaking for 10 min at room temperature, samples were centrifuged again at 15,000 rpm, 4 °C for 15 min. After removing the organic solvent layer and precipitated proteins, supernatants were leached with a 0.2-μm syringe filter. The filtered supernatants were then analyzed by tandem mass spectrometry (Agilent 6490 QQQ Triple Quadrupole LC/MS System^®^, Agilent Technologies, Santa Clara, CA) to measure MMC drug concentrations.

Oxaliplatin solutions were analyzed by inductively coupled plasma-quadrupole mass spectrometry (ICP-QMS; NexION 300D^®^, PerkinElmer, US). To measure platinum complex in the oxaliplatin, samples were prepared using a microwave digestion system. Samples were placed in Teflon vessels with 5 mL of HNO_3_ and digested in a microwave oven at 800 W for 1 h. After diluting to 25 mL with distilled water, samples were analyzed by ICP-QMS.

### Statistical Analyses

Statistical analyses were performed using SPSS 23 (SPSS Inc., Chicago, IL), SAS 9.3 (SAS Institute Inc., Cary, NC), and R 3.4.1 software (The R Foundation for Statistical Computing). Linear mixed models were used to compare drug concentrations between carrier solutions according to time. The independent *t* test and one-way analysis of variance (ANOVA) were used to compare area under the curve (AUC) ratios among carrier solutions. Post-hoc analyses were performed using the Scheffe correction method. A *p* value < 0.05 was considered statistically significant.

## Results

### Comparison of Drug Concentrations over Time During HIPEC

**Fig. 2:**
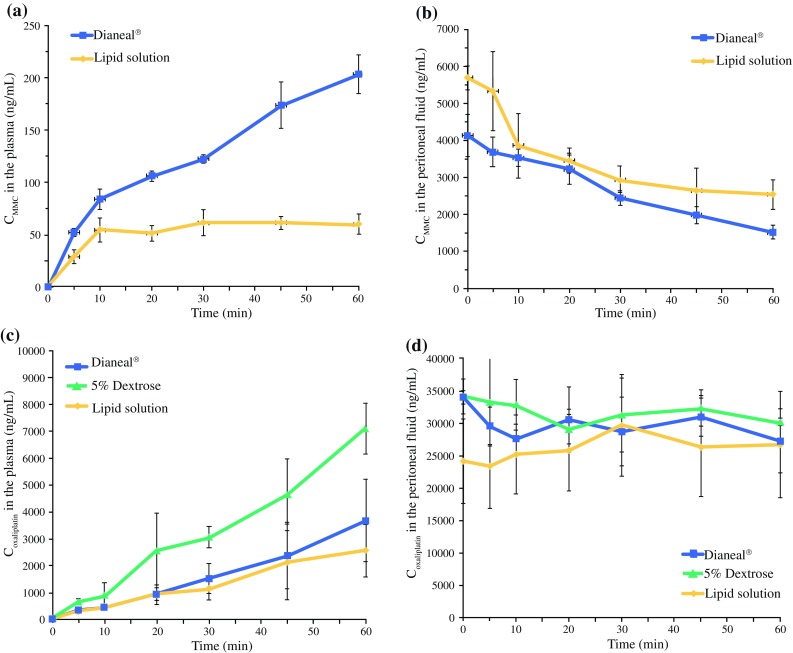
Concentrations of mitomycin-C (MMC) and oxaliplatin by HIPEC carrier solution. The concentration of MMC in plasma (**a**) and in the peritoneal fluid (**b**). The concentration of oxaliplatin in the plasma (**c**) and in the peritoneal fluid (**d**). *C*_MMC_, concentration of MMC; *C*_oxaliplatin_, concentration of oxaliplatin

In the MMC group, the plasma drug concentration in the Dianeal^®^ group was increased compared to the lipid solution over time, as shown in Table 1 and Fig. 2a. However, MMC concentrations in the peritoneal fluid were not significantly different between Dianeal^®^ and lipid solutions (*p* = 0.2313).

**Table 1:** Concentration of anticancer drugs between water and lipid carrier solutions during HIPEC

Anticancer drugs	Mitomycin-C					
Estimated samples	Plasma			Peritoneal fluid		
Carrier solutions	Drug concentration (ng/mL)		Overall *p* value^†^	Drug concentration (ng/mL)		Overall *p* value^†^
Time	Dianeal^®^ (*n* = 3)	Lipid solution (*n* = 3)		Dianeal^®^ (*n* = 3)	Lipid solution (*n* = 3)	
0	0 ± 0	0 ± 0	Carrier solution: *p* = 0.0419 Time: *p* < 0.0001 Carrier solution · time: *p* = 0.0332	4129.5 ± 562.1	5637.4 ± 353.7	Carrier solution: *p* = 0.1447 Time: *p* < 0.0001 Carrier solution · time: *p* = 0.2313
5	52 ± 4.0	33.5 ± 0.1		3687.6 ± 399.5	5110.3 ± 1190.9	
10	83.8 ± 9.5	54.0 ± 14.1		3532.3 ± 229.7	3651.0 ± 946.7	
20	105.9 ± 5.1	54.2 ± 5.7		3234.5 ± 421.0	3372.3 ± 362.2	
30	122.3 ± 3.8	58.1 ± 13.0		2440.7 ± 197.0	2776.2 ± 318.0	
45	173.8 ± 22.2	61.1 ± 6.1		1982.2 ± 233.5	2380.9 ± 351.0	
60	203.3 ± 18.7	60.0 ± 9.7		1515.8 ± 178.7	2366.9 ± 204.3	

Anticancer drugs	Oxaliplatin							
Estimated samples	Plasma				Peritoneal fluid			
Carrier solutions	Drug concentration (ng/mL)			Overall *p* value^†^	Drug concentration (ng/mL)			Overall *p* value^†^
Time	Dianeal^®^ (*n* = 3)	5% Dextrose (*n* = 3)	Lipid solution (*n* = 3)		Dianeal^®^ (*n* = 3)	5% Dextrose (*n* = 3)	Lipid solution (*n* = 3)	
0	0.0 ± 0.0	0.0 ± 0.0	0.0 ± 0.0	Carrier solution: *p* = 0.0048 Time: *p* < 0.0001 Carrier solution · time: *p* = 0.0049	34,053.1 ± 1020.0	34172.5 ± 2721.1	24,162.1 ± 6562.2	Carrier solution: *p* = 0.0307 Time: *p* = 0.7322 Carrier solution · time: *p* = 0.6249
5	331.3 ± 3.8	633.2 ± 121.6	276.2 ± 52.0		29,652.3 ± 2882.0	33,323.1 ± 6834.2	23,444.7 ± 6613.9	
10	433.2 ± 121.0	892.4 ± 447.7	440.4 ± 112.0		27,671.9 ± 2273.3	32,907.0 ± 3919.8	25,220.9 ± 6078.8	
20	928.5 ± 240.1	2548.6 ± 1406.8	906.7 ± 363.5		30,573.2 ± 5042.3	29,145.2 ± 2315.7	25,870.7 ± 6310.5	
30	1503.9 ± 555.1	3045.5 ± 397.9	1129.6 ± 428.9		28,737.9 ± 5267.0	31,320.5 ± 5696.8	29,700.3 ± 7873.8	
45	2356.9 ± 1238.4	4627.5 ± 1346.4	2126.0 ± 1403.9		30,996.5 ± 2960.2	32,412.8 ± 2838.9	26,504.4 ± 7815.0	
60	3667.1 ± 1527.2	7093.9 ± 946.1	2541.9 ± 966.1		27,329.7 ± 4951.2	30,239.3 ± 598.2	26,755.2 ± 8213.6	

Mean ± standard deviation

^†^The linear mixed model was used to calculate *p* values and to compare the concentration of anticancer drugs among carrier solutions according to the time

In the oxaliplatin group, the plasma drug concentrations were significantly different among the carrier solutions by time (*p* = 0.0049). The plasma absorption rate was highest in the oxaliplatin mixed with 5% dextrose solution group (Fig. 2c). However, oxaliplatin concentrations in the peritoneal fluid were not significantly different among the carrier solutions (Table 1).

### Comparison of AUC Ratios Between Water and Lipid Solutions

In the MMC group, the AUC of the peritoneal fluid (AUC_peritoneum_) was higher in the lipid carrier solution than Dianeal^®^. Conversely, the AUC of the plasma (AUC_plasma_) was lower in the lipid solution compared to Dianeal^®^. Thus, the peritoneal/plasma AUC ratio of the lipid carrier solution was approximately three times higher than Dianeal^®^ in the MMC group (*p* < 0.001; Table 2).

**Table 2:** Comparison of AUC ratios between water and lipid carrier solutions during HIPEC

Anticancer drugs	Mitomycin-C			Oxaliplatin						
Carrier solutions	Dianeal^®^	Lipid solution	*p* value^†^	Dianeal^®^	5% dextrose solution (5DW)	Lipid solution	*p* value^††^	Post-hoc analysis^a^		
								*p* value (Dianeal vs. 5DW)	*p* value (Dianeal vs. lipid)	*p* value (5DW vs. lipid)
AUC_peritoneum_	159,209.3 ± 6843.8	188,918.4 ± 3341.9	**0.003**	1,671,286 ± 176,313.1	1,690,563 ± 411,394.1	1,594,976 ± 418,625.8	0.941	0.998	0.966	0.947
AUC_plasma_	7684.8 ± 575.4	3203.1 ± 46.4	**0.005**	95,540.1 ± 39,851.3	196,029.4 ± 47,823.8	78,824.3 ± 37,743.6	**0.028**	0.070	0.890	**0.039**
AUC ratio^b^	20.8 ± 1.6	59.0 ± 1.0	**<** **0.001**	19.3 ± 6.6	8.7 ± 1.6	22.2 ± 6.1	**0.046**	0.125	0.806	**0.056**

Bold values are statistically significant (*p* < 0.05)

^†^Independent *t* test

^††^One-way analysis of variances among groups (ANOVA)

^a^Post-hoc analysis was calculated by the Scheffe correction method

^b^AUC_peritoneum_/AUC_plasma_

In the oxaliplatin group, the AUC_peritoneum_ was not significantly different among the carrier solutions (*p* = 0.941). However, the AUC_plasma_ of the 5% dextrose solution was higher than that of the lipid solution (*p* = 0.039). There was no significant difference in the AUC_plasma_ between the 5% dextrose solution and Dianeal^®^ (*p* = 0.070). The oxaliplatin AUC ratio was different among the carrier solutions. In particular, the oxaliplatin AUC ratio in the lipid solution was marginally higher than that of the 5% dextrose solution during HIPEC (*p* = 0.056). According to AUC ratios of oxaliplatin, which were cut off at 30 min, AUC ratio of lipid solution was higher than 5% dextrose solution (Supplementary Table 1).

### Plasma Drug Concentration Gradient by Carrier Solution

To estimate the changes in plasma drug concentration among the carrier solutions, the plasma concentration gradient of anticancer drugs was calculated using a linear mixed model, according to time. As shown in Table 3, the estimated plasma concentration gradient of MMC had a steeper slope with Dianeal^®^ compared with the lipid solution. On the other hand, the estimated plasma concentration gradient of oxaliplatin had a steeper slope in the 5% dextrose solution compared to both the Dianeal^®^ and the lipid solution (*p* < 0.001). However, there was no significant difference in the oxaliplatin estimated plasma concentration gradient between the Dianeal^®^ and lipid solutions.

**Table 3:** Estimated formulas for the anticancer drug concentration in the plasma

Anticancer drugs	Carrier solutions	Estimated formulas of the graph for the plasma drug concentrations (MMC: Fig. 2a, oxaliplatin: Fig. 2c)	Estimated concentration gradient (SE)	*p* value^†^
Mitomycin-C	Dianeal^®^	*C*_plasma_ = 3.05 · time + 30.99	3.05 (0.21)	**<** **0.0001**
	Lipid solution	*C*_plasma_ = 0.74 · time + 27.80	0.74 (0.23)	
Oxaliplatin	Dianeal^®^	*C*_plasma_ = 59.15 · time − 126.21	59.15 (7.52)	**<** **0.0001** (Dianeal vs. 5DW, *p* < 0.001; Dianeal vs. lipid, *p* = 0.3957; 5DW vs. lipid, *p* < 0.001)
	5% dextrose solution	*C*_plasma_ = 113.21 · time − 57.74	113.21 (7.35)	
	Lipid solution	*C*_plasma_ = 43.05 · time + 14.56	43.05 (7.35)	

Estimated formulas was calculated by random intercept model

Bold values are statistically significant (*p* < 0.05)

*C*_plasma_, concentration of plasma; SE, standard error; 5DW, 5% dextrose

^†^Calculated by linear mixed model

## Discussion

HIPEC has pharmacologic principles that provide regionally intensified antineoplastic drug concentration in the peritoneal cavity and promote tumor cell penetration with a prolonged presence in the peritoneal-plasma barrier.^3^ Based on the anatomical structures of the peritoneum, anticancer drugs that have large MW and water insolubility are correlated with a larger AUC ratio and longer stay in the peritoneal cavity.^1^ The intercellular gaps of the mesothelium are larger than those in the endothelium; therefore, large molecules that cannot pass through endothelial layers do penetrate mesothelial layers. The MW of MMC is 334.3 g/mol and that of oxaliplatin is 397.3 g/mol, both smaller than anticancer drugs paclitaxel (MW = 853.9 g/mol) and docetaxel (MW = 861.9 g/mol).^4^ In addition, the logarithm ratio of partition coefficient (log *P*) of MMC is − 1.6 and oxaliplatin is − 0.47, which tends to be water-soluble. Therefore, these pharmacologic characteristics of both MMC and oxaliplatin are not inherently suitable to enhance the effect of IP chemotherapy, with the exception of water solubility, which is a useful characteristic to circulate solutes during HIPEC.

However, importantly, our results demonstrate that the use of a lipid carrier solution increased the AUC ratio and reduced the plasma absorption rate. Although the oxaliplatin AUC_peritoneum_ was not significantly different among carrier solutions, our data showed that a lipid carrier solution has advantages, controlling the permeability of endothelial layers and reducing the plasma absorption rate during HIPEC. This could be a result of hydrophobic lipid particles that are resistant to traversing the plasma membrane of endothelial cell layers.

In HIPEC treatment of colorectal cancer patients with peritoneal carcinomatosis, both MMC and oxaliplatin are used. The MMC carrier solution currently used in most HIPEC centers is an isotonic solution.^15^ Therefore, in this study, we compared the effects of carrier solutions except 5% dextrose solution in the MMC HIPEC. However, the optimal selection of carrier solution during oxaliplatin HIPEC is still debated, as oxaliplatin can be degraded in chloride-containing carrier solutions, because the oxalate ligand of oxaliplatin can be substituted into chloride ions.^12^ Elias et al.^16^ reported favorable oncological outcomes using oxaliplatin with 5% dextrose carrier solution. However, it has been reported that the hypotonicity of the dextrose solution increases the risk of postoperative complications after HIPEC, such as severe electrolyte imbalance, hyperglycemia, tissue edema, and intraperitoneal hemorrhagic complications.^10^,^17^–^19^ Therefore, in the current study, we compared the oxaliplatin AUC ratios among three carrier solutions: 5% dextrose solution, Dianeal^®^, and lipid solution to compare lipophilicity versus hydrophilicity and chloride-containing versus non-chloride-containing solutions.

According to the comparison of carrier solutions between lipophilicity and hydrophilicity, the oxaliplatin AUC_peritoneum_ was not significantly different among carrier solutions. However, the AUC_plasma_ of the lipid solution was lower than that of the 5% dextrose solution. In addition, the AUC ratio in the lipid solution was marginally higher than hydrophilic carrier solution in both oxaliplatin and MMC HIPEC. Thus, in this study, the lipophilicity of a carrier solution seemed to have an advantage in reducing plasma absorption and increasing the AUC ratio compared with hydrophilic carrier solutions.

In HIPEC using oxaliplatin, 30-min duration is regarded as clinically suitable considering half-life of oxaliplatin and systemic toxicities.^20^ However, because this study was the first experiment to use a lipophilic carrier solution for oxaliplatin-HIPEC, HIPEC was performed to evaluate fully pharmacologic properties of lipid carrier solution until 60 min. The AUC ratio of oxaliplatin in the lipid solution was higher than 5% dextrose solution at both 30 and 60 min.

Compared with the pharmacological effects of oxaliplatin in the chloride-containing solutions, our results support the effectiveness of Dianeal^®^. The chloride concentration of Dianeal^®^ is 96 mmol/L, whereas that for 5% dextrose solution is 0 mmol/L. Lipision^®^ is composed of purified soybean oil, purified phospholipid, and glycerin. As demonstrated in Table 2, Dianeal^®^ exhibited advantages to reducing plasma absorption of anticancer drugs, compared with 5% dextrose solution, when performing oxaliplatin HIPEC. In addition, structural instability of oxaliplatin in the chloride-containing solutions during HIPEC could be acceptable, as a previous report by Mehta et al.^12^,^21^ indicated that the degradation rates of oxaliplatin is limited within 10–15% in the chloride-containing HIPEC carrier solution. It is also expected that peritoneal dialysis solutions such a Dianeal^®^ can have advantages to reduce postoperative complications, such as electrolyte imbalance and metabolic disturbance.

Our study results also showed that the AUC ratio of a lipid carrier solution was larger than other carrier solutions. However, there are some limitations in using lipid carrier solutions in clinical applications for HIPEC. Lipision^®^, which was used in this study, is a fat emulsion. Although the lipid layers of Lipision^®^ retard plasma absorption rates of anticancer drugs during HIPEC, the hypertonicity and electrical resistance of these lipid layers can inhibit the permeability of peritoneal-plasma barriers. In addition, according to the pharmacokinetic principles of HIPEC, longer duration in the peritoneum delays recirculation of the tumor core.^1^ Because the efficacy of HIPEC is related to sustained peritoneal drug concentrations, as well as drug infiltration into the tumor core, this contrary phenomenon should be considered in selecting an optimal IP chemotherapeutic agent. In our results, although the lipid carrier solution prolonged peritoneal occupancy, as well as reduced plasma absorption rate, the hydrophobicity of the lipid solution might be inadequate to recirculate into tumor core from the capillary vessels and to increase cytotoxicity. Furthermore, because the bioavailability of anticancer agents is assessed from the release rate of an entrapped drug in lipid layers, it can be questioned whether anticancer agents mixed in a lipid carrier solution have complete tumor cell cytotoxicity during HIPEC.^3^,^22^,^23^ The release rate of anticancer drugs in the lymphatic channels and the risk of fat embolism are also to be considered when a lipid carrier solution is used during HIPEC.

This study has several limitations, including a small sample-sized experiment in the animal model. In addition, there is a lack of investigation into both the cytotoxic effects and the rate of lymphatic spread of anticancer drugs during HIPEC with a lipophilic carrier solution. Naïve oxaliplatin is known to have less cytotoxicity than dichloro-platinum compound Pt(dach)Cl_2_, which is an active form that is transformed in chloride-containing media.^24^,^25^ Because our study measured platinum concentration of oxaliplatin, further studies are required to measure the concentration of oxaliplatin transformation to understand both structural instability and cytotoxicity of oxaliplatin depending on different HIPEC carrier solutions.

## Conclusions

A lipid carrier solution is promising, because it increases the AUC ratio and decreases plasma absorption during HIPEC. However, 5% dextrose solution is inferior to both Dianeal^®^ and lipid solutions as an oxaliplatin-based HIPEC carrier solution. The choice between Dianeal^®^ and lipid solutions should be made based on considerations of safety and further data regarding the actual efficacy of cytotoxic agents in these solutions.

## Electronic supplementary material

Below is the link to the electronic supplementary material.

Supplementary material 1 (DOCX 14 kb)
