# Supplementary material for: Pharmacologic Properties of the Carrier Solutions for Hyperthermic Intraperitoneal Chemotherapy: Comparative Analyses Between Water and Lipid Carrier Solutions in the Rat Model
Source: Ann Surg Oncol. 2018 Jul 18;25(11):3185–92. doi: 10.1245/s10434-018-6628-x (PMC6132421; doi:10.1245/s10434-018-6628-x)
Supplement: Supplementary file 1 — Supplementary material 1 (DOCX 14 kb) [file 10434_2018_6628_MOESM1_ESM.docx]

Supplementary Table 1. Comparison of AUC ratios of oxaliplatin during 30 minutes of HIPEC

| Anticancer drugs | Oxaliplatin | | | | | | |
| --- | --- | --- | --- | --- | --- | --- | --- |
| Carrier solutions | **Dianeal^®^** | **5% Dextrose solution (5DW)** | **Lipid solution** | **P value^††^** | **Post-hoc analysis^§^** | | |
|  |  |  |  |  | **P value**  **(Dianeal vs. 5DW)** | **P value**  **(Dianeal vs. Lipid)** | **P value**  **(5DW vs. Lipid)** |
| AUC_peritoneum_ | 775502.7±137773 | 904413.1±160077.2 | 776209.1±192779.2 | 0.576 | 0.653 | >0.9999 | 0.656 |
| AUC_plasma_ | 21092.3±5986.7 | 49536.9±18242.4 | 18945.4±6092.5 | **0.032** | 0.064 | 0.975 | **0.049** |
| AUC ratio^*^ | 38.3±10.2 | 20.2±8.2 | 42.0±5.6 | **0.040** | 0.102 | 0.850 | **0.051** |

AUC ratio^*^= AUC_peritoneum_/AUC_plasma_; **^†^**Independent t-test; **^††^**One-way analysis of variances among groups (ANOVA); **^§^**Post-hoc analysis was calculated by the Scheffe correction method.
